# Supplementary material for: Caterpillar-induced rice volatiles provide enemy-free space for the offspring of the brown planthopper
Source: eLife. 2020 Aug 11;9:e55421. doi: 10.7554/eLife.55421 (PMC7419140; doi:10.7554/eLife.55421)
Supplement: Figure 4—source data 1. — Volatile compounds collected from the headspace of control rice plants (Uninfested), plants infested with 10 gravid BPH females for 12 hr (Infested by BPH (10)), plants infested with two 3rd-instar SSB larvae for 12 hr and then 10 gravid BPH females for another 12 hr (Infested by SSB plus BPH (2:10)), plants infested with two SSB larvae for 12 hr and then five gravid BPH females for another 12 hr (Infested by SSB plus BPH (2:5)), and plants infested with two SSB larvae for 24 hr (Infested by SSB (2)). The values represent the mean percentages ± SE of the peak area relative to the peak area of the internal standard (nonyl acetate). Letters following each value in the same row indicate significant differences between rice plant treatments based on one-way ANOVA followed Tukey HSD test (p<0.05) (N = 7–8). Data were fourth-root transformed before analysis. [file elife-55421-fig4-data1.docx]

**Figure 4-source data 1:** **Volatile compounds released by differently infested rice plants.** Volatile compounds collected from the headspace of control rice plants (Uninfested), plants infested with 10 gravid BPH females for 12 h (Infested by BPH (10)), plants infested with two 3^rd^-instar SSB larvae for 12 h and then 10 gravid BPH females for another 12 h (Infested by SSB plus BPH (2:10)), plants infested with two SSB larvae for 12 h and then 5 gravid BPH females for another 12 h (Infested by SSB plus BPH (2:5)), and plants infested with two SSB larvae for 24 h (Infested by SSB (2)). The values represent the mean percentages ± SE of the peak area relative to the peak area of the internal standard (nonyl acetate). Letters following each value in the same row indicate significant differences between rice plant treatments based on one-way ANOVA followed Tukey HSD test (*P* < 0.05) (N = 7-8). Data were fourth-root transformed before analysis.

| **Peak no.** | **Plant volatile** | **Retention time (min)** | **Uninfested** | | **Infested by BPH (10)** | **Infested by SSB plus BPH (2:10)** | **Infested by SSB plus BPH (2:5)** | **Infested by SSB**  **(2)** | ***P*** | |
| --- | --- | --- | --- | --- | --- | --- | --- | --- | --- | --- |
| 1 | (*Z*)-3-hexenal | 4.437 | 3.81±0.26b | 9.79±0.30b | | 26.07±2.92a | 31.97±6.36a | 32.72±8.51a | | **<0.001** |
| 2 | hexanal | 4.483 | 12.03±1.88b | 19.01±4.09b | | 52.49±10.02a | 49.50±8.83a | 29.88±4.05ab | | **<0.001** |
| 3 | (*E*)-2-hexenal | 5.666 | 3.53±0.42c | 26.37±14.01bc | | 125.13±46.82a | 137.62±42.07a | 61.52±19.21ab | | **<0.001** |
| 4 | p-xylene | 6.028 | 13.73±2.36 | 25.26±7.39 | | 34.64±8.72 | 32.92±7.51 | 34.48±9.67 | | 0.148 |
| 5 | 2-heptanone | 6.554 | - | 21.41±4.50b | | 83.87±22.08a | 95.58±14.24a | 142.47±26.84a | | **<0.001** |
| 6 | 2-heptanol | 6.887 | 6.85±1.55c | 9.55±3.19c | | 36.36±8.69b | 48.40±5.83ab | 82.51±19.95a | | **<0.001** |
| 7 | α-pinene | 7.644 | 4.89±0.98b | 14.81±4.94a | | 10.52±2.45a | 13.20±1.50a | 14.37±2.95a | | **0.019** |
| 8 | mesitylene | 9.295 | 3.52±0.41 | 3.85±0.47 | | 3.55±0.12 | 3.33±0.31 | 3.56±0.51 | | 0.94 |
| 9 | octanal | 9.572 | 12.43±2.65 | 8.73±1.20 | | 12.32±1.85 | 12.81±1.44 | 13.67±1.25 | | 0.284 |
| 10 | o-cymene | 10.145 | 1.50±0.46b | 1.97±026ab | | 2.66±0.51ab | 3.32±0.40a | 3.98±0.58a | | **0.011** |
| 11 | D-limonene | 10.274 | 7.80±2.48b | 11.06±2.05b | | 36.38±5.18a | 50.57±5.38a | 46.12±7.50a | | **<0.001** |
| 12 | acetophenone | 11.256 | 4.86±0.62 | 4.44±0.68 | | 4.55±0.82 | 4.58±0.66 | 5.50±0.61 | | 0.789 |
| 13 | (*E*)-2-heptenyl acetate | 11.383 | - | - | | 10.45±1.37 | 11.45±0.82 | 14.49±1.66 | | 0 .121 |
| 14 | 2-nonanone | 11.956 | 3.43±0.32c | 3.55±0.86c | | 16.13±3.68b | 22.81±4.41ab | 34.88±7.82a | | **<0.001** |
| 15 | benzoic acid, methyl ester | 12.046 | 2.21±1.01c | 2.44±0.39c | | 5.26±0.52bc | 6.77±1.10ab | 9.52±1.10a | | **<0.001** |
| 16 | linalool | 12.234 | 58.26±10.61b | 100.32±36.21ab | | 77.78±10.65ab | 94.08±8.86ab | 142.72±20.17a | | **0** **.025** |
| 17 | nonanal | 12.329 | 29.95±6.01 | 19.05±2.78 | | 27.06±3.11 | 27.08±5.77 | 32.02±2.59 | | 0.150 |
| 18 | DMNT^a^ | 12.571 | - | 4.36±0.52b | | 5.77±1.40ab | 6.60±0.69ab | 9.54±1.98a | | **0** **.017** |
| 19 | isophorone | 12.787 | 3.49±1.00b | 6.32±1.08ab | | 7.91±2.02ab | 7.74±1.24ab | 12.85±2.60a | | **0.010287** |
| 20 | naphthalene | 14.457 | 3.90±0.47 | 3.33±0.63 | | 3.50±0.31 | 3.54±0.43 | 4.71±0.46 | | 0.345 |
| 21 | methyl salicylate | 14.654 | 5.75±1.78b | 7.82±2.88ab | | 7.56±1.24ab | 10.95±1.66ab | 18.83±4.05a | | **0.037** |
| 22 | 2,4-dimethylbenzaldehyde | 15.256 | 4.44±0.28 | 5.03±0.70 | | 4.11±0.46 | 4.99±0.29 | 4.45±0.55 | | 0. 609 |
| 23 | unknown 1 | 16.394 | 8.39±2.39 | 8.10±1.15 | | 6.61±6.87 | 6.87±0.98 | 10.09±2.77 | | 0.584 |
| 24 | 2-undecanone | 17.158 | 1.28±0.46c | 2.56±0.50bc | | 3.43±0.40bc | 5.03±0.86ab | 7.91±1.70a | | **<0.001** |
| 25 | unknown 2 | 17.274 | 2.74±0.36 | 2.20±0.63 | | 2.32±0.22 | 2.50±0.27 | 2.93±0.89 | | 0. 782 |
| 26 | α-copaene | 19.211 | - | 0.56±0.11b | | 3.81±0.72a | 6.44±0.88a | 5.05±0.74a | | **<0.001** |
| 27 | tetradecane | 19.716 | 6.08±1.16 | 4.19±0.70 | | 5.35±0.64 | 5.04±0.73 | 5.95±0.42 | | 0. 826 |
| 28 | longifolene | 20.024 | 3.51±0.40 | 1.86±0.42 | | 2.51±0.55 | 2.29±0.43 | 3.77±0.61 | | 0.054 |
| 29 | (*E*)-β-caryophyllene | 20.233 | 1.04±0.13b | 1.75±0.17ab | | 1.99±0.39ab | 3.24±0.48a | 3.04±0.40a | | **<0.001** |
| 30 | (*E*)-α-bergamotene | 20.499 | - | - | | 2.17±0.61 | 2.70±0.37 | 2.95±0.33 | | 0.316 |
| 31 | unknown 3 | 20.658 | - | 0.76±0.11b | | 1.57±0.27a | 1.93±0.25a | 2.16±0.22a | | **0** **.004** |
| 32 | 2,6,10-trimethyltridecane | 21.066 | 1.97±0.29 | 1.49±0.13 | | 1.77±0.16 | 2.20±0.30 | 2.42±0.27 | | 0.125 |
| 33 | unknown 4 | 21.488 | - | 1.45±1.45b | | 2.98±0.69ab | 4.59±1.40a | 2.24±0.35ab | | 0.039 |
| 34 | germacrene D | 21.596 | - | - | | 2.97±0.63 | 3.71±0.54 | 3.41±0.52 | | 0 .718 |
| 35 | 2-tridecanone | 21.807 | - | 5.80±1.63b | | 6.37±1.24b | 10.55±1.88ab | 13.76±2.86a | | **0** **.032** |
| 36 | pentadecane | 21.941 | 10.70±1.48 | 8.20±1.05 | | 9.37±0.84 | 10.11±1.24 | 11.14±0.86 | | 0 .483 |
| 37 | tridecanal | 22.158 | 4.61±0.88b | 4.18±0.83b | | 8.96±2.05ab | 13.08±2.05a | 11.34±1.18a | | **0.002** |
| 38 | unknown 5 | 22.383 | - | - | | 2.05±0.34 | 3.53±0.64 | 3.26±0.42 | | 0.089 |
| 39 | cubenene | 22.697 | - | - | | 1.97±0.51b | 5.32±1.11a | 2.28±0.34b | | **0.035** |
| 40 | unknown 6 | 23.461 | - | 5.27±1.03 | | 3.56±0.50 | 2.94±0.72 | 3.53±0.47 | | 0 .320 |
| 41 | TMTT^b^ | 23.485 | - | - | | 2.95±0.51 | 2.02±0.36 | 2.53±0.61 | | 0 .252 |
| 42 | unknown 7 | 23.773 | 6.46±2.93 | 7.87±1.34 | | 9.88±1.52 | 11.84±2.75 | 14.09±1.81 | | 0 .149 |
| 43 | hexadecane | 24.041 | 10.91±1.98 | 8.76±1.07 | | 10.45±0.98 | 10.54±1.52 | 11.82±0.90 | | 0 .688 |
| 44 | cedrol | 24.385 | 0.88±0.18b | 2.56±0.48ab | | 2.94±0.53a | 2.48±0.98ab | 3.04±0.66a | | **0** **.014** |
| 45 | 2,6,10-trimethylpentadecane | 24.986 | 6.30±1.02 | 4.74±0.53 | | 5.46±0.73 | 5.72±0.98 | 7.40±0.77 | | 0 .455 |
| 46 | 2-pentadecanone | 25.973 | - | 2.49±0.65b | | 5.86±1.74a | 7.34±1.66a | 8.31±1.45a | | **0** **.004** |
| 47 | heptadecane | 26.036 | 16.19±2.07 | 12.75±1.64 | | 14.12±1.40 | 15.80±3.42 | 16.25±1.36 | | 0 .802 |
| 28 | benzyl benzoate | 27.368 | 12.87±2.90 | 13.44±3.50 | | 13.45±2.03 | 15.07±4.22 | 12.18±1.66 | | 0 .994 |
| 49 | anthracene | 27.695 | 5.71±0.98 | 4.81±0.89 | | 5.79±0.50 | 5.87±1.19 | 5.82±0.62 | | 0 .831 |
| 50 | nonadecane | 27.937 | 5.50±0.60 | 4.66±0.49 | | 5.10±0.52 | 5.05±0.77 | 5.59±0.42 | | 0 .801 |
| 51 | isopropyl myristate | 28.346 | 40.88±7.11b | 62.27±3.08ab | | 91.32±12.95ab | 124.74±28.61a | 117.22±19.12a | | **0** **.013** |
| 52 | unknown 8 | 28.509 | 3.94±0.84 | 3.08±1.24 | | 3.53±0.67 | 3.56±0.99 | 4.09±0.42 | | 0 .724 |
| 53 | unknown 9 | 28.977 | 5.92±0.76 | 6.18±1.12 | | 4.75±1.31 | 4.92±0.86 | 5.80±0.62 | | 0 .485 |
| 54 | homosalate | 29.544 | 2.59±0.46 | 2.15±0.34 | | 2.49±0.41 | 2.19±0.45 | 2.32±0.23 | | 0. .883 |
| 55 | heneicosane | 31.454 | 2.05±0.15a | 1.78±0.13ab | | 1.47±0.10b | 1.51±0.16b | 1.83±0.26ab | | **0 .037** |

^a^ DMNT = (3*E*)-4,8-dimethyl-1,3,7-nonatriene.

^b^ TMTT **=** (*3E,7E*)-4,8,12-trimethyl-1,3,7,11-tridecatetraene.

“-” indicates that the concentration of the volatile was below the detection level.

“unknowns” were the compounds that were not confirmed with authentic standards.
