# Supplementary material for: Caterpillar-induced rice volatiles provide enemy-free space for the offspring of the brown planthopper
Source: eLife. 2020 Aug 11;9:e55421. doi: 10.7554/eLife.55421 (PMC7419140; doi:10.7554/eLife.55421)
Supplement: Figure 6—source data 1. — Volatile compounds were mixed in pure hexane in ratios that correspond to the ratios of compounds detected in the collection of volatiles from insect infested rice plants. The obtained hexane solutions were used to test the responses of female Anagrus nilaparvatae wasps to each synthetic blend. [file elife-55421-fig6-data1.docx]

**Figure 6-source data 1: Concentrations of 13 volatile compounds contained in each synthetic blend.** Volatile compounds were mixed in pure hexane in ratios that correspond to the ratios of compounds detected in the collection of volatiles from insect infested rice plants. The obtained hexane solutions were used to test the responses of female *Anagrus nilaparvatae* wasps to each synthetic blend.

| No. | Compounds | Synthetic blends (ng/10 μl hexane) | | | |
| --- | --- | --- | --- | --- | --- |
|  |  | 10 BPH | 2 SSB + 10 BPH | 2 SSB + 5BPH | 2 SSB |
| 1 | (*E*)-2-hexenal | 81 | 384 | 422 | 189 |
| 2 | 2-heptanol | 13 | 51 | 68 | 116 |
| 3 | α-pinene | 22 | 16 | 20 | 22 |
| 4 | D-limonene | 16 | 52 | 72 | 66 |
| 5 | 2-nonanone | 5 | 22 | 31 | 48 |
| 6 | linalool | 160 | 124 | 150 | 228 |
| 7 | DMNT | 12 | 16 | 18 | 27 |
| 8 | (*E*)-2-heptenyl acetate | 0 | 14 | 15 | 19 |
| 9 | methyl salicylate | 18 | 18 | 26 | 44 |
| 10 | (*E*)-β-caryophyllene | 2 | 2 | 4 | 4 |
| 11 | 2-tridecanone | 6 | 6 | 11 | 14 |
| 12 | TMTT | 0 | 3 | 2 | 2 |
| 13 | isopropyl myristate | 46 | 67 | 92 | 87 |
